# Supplementary material for: Analysis of chemical exchange in iridium N-heterocyclic carbene complexes using heteronuclear parahydrogen-enhanced NMR
Source: Commun Chem. 2024 Dec 3;7:286. doi: 10.1038/s42004-024-01376-z (PMC11614900; doi:10.1038/s42004-024-01376-z)
Supplement: Supplementary file 2 — Supplementary Information [file 42004_2024_1376_MOESM2_ESM.pdf]

Supplementary Information

Analysis of Chemical Exchange in Iridium N-Heterocyclic Carbene Complexes Using Heteronuclear Parahydrogen-Enhanced NMR

Charbel D. Assaf[a]*, Xin Gui[b], Oleg G. Salnikov[c], Arne Brahms[d], Nikita V. Chukanov[c], Ivan V. Skovpin[c], Eduard Y. Chekmenev[e], Rainer Herges[d], Simon B. Duckett[f], Igor V. Koptyug[c], Kai Buckenmaier[g], Rainer Körber[h], Markus Plaumann[i], Jan-Bernd Hövener[a], Alexander Auer[b], Andrey N. Pravdivtsev[a]*

[a] Section Biomedical Imaging, Molecular Imaging North Competence Center (MOIN CC), Department of Radiology and Neuroradiology, University Medical Center Kiel, Kiel University, Am Botanischen Garten 14, 24118, Kiel, Germany

[b] Max-Planck-Institut für Kohlenforschung, Kaiser-Wilhelm-Platz 1, 45470 Mülheim an der Ruhr

[c] International Tomography Center SB RAS, 3A Institutskaya St., 630090 Novosibirsk, Russia

[d] Otto Diels Institute for Organic Chemistry, Kiel University, Otto- Hahn Platz 4, 24118 Kiel, Germany

[e] Department of Chemistry, Integrative Biosciences (Ibio), Karmanos Cancer Institute (KCI), Wayne State University, Detroit, MI 48202, United States

[f] Centre for Hyperpolarization in Magnetic Resonance (CHyM), University of York, Heslington YO10 5NY, UK

[g] High-Field Magnetic Resonance Center, Max Planck Institute for Biological Cybernetics, Max-Planck-Ring 11, 72076 Tübingen, Germany

[h] Physikalisch-Technische Bundesanstalt (PTB), Abbestraße 2-12, 10587 Berlin, Germany

[i] Otto-von-Guericke-University Magdeburg Institute for Molecular Biology and Medicinal Chemistry, Leipziger Str. 44, 39120 Magdeburg, Germany

Contents

Supplementary Methods: Calibration of variable temperature NMR unit 3

Supplementary Methods: Signal enhancement and polarization calculations...................................... 4

Supplementary Methods: Weighted mean k_d.. 4

Supplementary Methods: Fitting of exchange rate constants as a function of temperature 5

Supplementary Methods: ^{15}N chemical shifts of ligands and comparison of ESOTHERIC and phINEPT+ SOT performance... 5

Supplementary Discussion: Phase distortion in the ESOTHERIC SOT sequence 6

Supplementary Discussion: Exchange rate constants measured with SABRE-ESOTHERIC 7

Supplementary Discussion: Comparison of exchange rate constants with literature data 13

Supplementary Discussion: Enthalpies and entropies of activation 14

Supplementary Discussion: Analysis of SABRE complexes with metronidazole 16

Supplementary Discussion: N.a. pyridine exchange 19

Supplementary Discussion: Quantum chemical calculations... 20

Supplementary References: 22

Supplementary Methods: Calibration of variable temperature NMR unit

We calibrated the variable temperature unit (VTU) of NMR spectrometer using a common method of observing the chemical shift separation Δ between the OH and CH_3 resonances of methanol. We prepared 600 µL of 4:96 mixture of isotopically unlabelled methanol and methanol-d_4 and measured its ^1H NMR spectra at different temperatures. Then we used the following two equations to calculate the actual temperature of the sample as in the Bruker Instruments Manual for VT-Calibration:

1) For values between 230 – 270 K:

$$T = (3.92 - \Delta)/0.008 \qquad \textit{(Eq. S1)}$$

2) For more accurate values between 270 – 300 K we used this equation:

$$T = (4.109 - \Delta)/0.008708 \qquad \textit{(Eq. S2)}$$

Table S1. The nominal NMR temperature set by the user and the calibrated temperature using Eqs. S1 and S2. Additionally, we showed the relative deviation between the calibrated and the nominal temperatures.

Nominal NMR T (K)	Calibrated T (K)	Deviation (%)
260	257.6	−0.92
262.5	260.3	−0.84
267	265.2	−0.67
270	268.4	−0.059
273	272	−0.37
275	273.84	−0.44
277	276	−0.36
280	279.3	−0.25
283	282.5	−0.18
288	287.9	−0.035
293	293.4	+0.14

Supplementary Methods: Signal enhancement and polarization calculations

Signal enhancement

The enhancement factor ε of equatorial substrates was calculated using the signal intensities of the spectra of thermally polarized and hyperpolarized solutions, considering the differences in acquisition parameters. The signal intensities of the free and equatorial-bound substrate in the proton spectrum were used to assess the ratio of concentrations of these two forms in a case when the equatorial substrate peak in the thermal ^{15}N spectra was negligible compared to the noise level. The following formula was used

$$\varepsilon = \frac{{}^{15\mathrm{N}}I_{\mathrm{e}}^{\mathrm{HP}}}{{}^{15\mathrm{N}}I_{\mathrm{f}}^{\mathrm{TP}}} \times \frac{{}^{1\mathrm{H}}I_{\mathrm{f}}^{\mathrm{TP}}}{{}^{1\mathrm{H}}I_{\mathrm{e}}^{\mathrm{TP}}} \times \frac{\sin(\alpha_{\mathrm{TP}})}{\sin(\alpha_{\mathrm{HP}})} \times \frac{RG_{\mathrm{TP}}}{RG_{\mathrm{HP}}} \times \frac{NS_{\mathrm{TP}}}{NS_{\mathrm{HP}}} \quad \textit{(Eq. S3)}$$

where ${}^{15\mathrm{N}}I_{\mathrm{e}}^{\mathrm{HP}}$ is the ^{15}N integral of the hyperpolarized equatorial-bound substrate, ${}^{15\mathrm{N}}I_{\mathrm{f}}^{\mathrm{TP}}$ is the ^{15}N integral of the thermally polarized free substrate, ${}^{1\mathrm{H}}I_{\mathrm{f}}^{\mathrm{TP}}$ is the ^{1}H integral of the free substrate at thermal equilibrium, ${}^{1\mathrm{H}}I_{\mathrm{e}}^{\mathrm{TP}}$ is the ^{1}H integral of the equatorial-bound substrate at thermal equilibrium. NS_{TP} and NS_{HP} are the numbers of scans for the thermally polarized and hyperpolarized samples, and α_{TP} and α_{HP} are the excitation flip angles of RF pulses used to acquire the corresponding spectra. RG_{TP} and RG_{HP} are values of linear receiver gain.

Polarization

To compare the enhancements at 9.4 T more easily, the enhancement factors ε were converted to a polarization level, P, using Eq. S2, where, γ is the gyromagnetic ratio, B_0 is the detection field, T is the temperature, ħ is the reduced Planck's constant, and k_B is Boltzmann's constant.

$$P = \varepsilon \frac{\gamma B_0 \hbar}{2\, k_{\mathrm{B}} T} \quad \textit{(Eq. S4)}$$

Supplementary Methods: Weighted mean k_d

We computed the weighted mean k_d values considering the uncertainties associated with three distinct measurements. This approach allowed us to account for our dataset's varying uncertainty levels. Subsequently, we derived the combined standard deviation of the weighted mean k_d, providing an overall variability in our data.

If values are x_i, and the corresponding standard deviations are s_{xi}, then the combined standard deviation is

$$s_{\bar{x}} = \frac{1}{\sqrt{\left(\sum_i \frac{1}{s_{xi}^2}\right)}}$$

(Eq. S5)

And the corresponding weighted mean is:

$$\bar{x} = \left(\sum_i \frac{x_i}{s_{xi}^2}\right) s_{\bar{x}}^2$$

(Eq. S6)

Supplementary Methods: Fitting of exchange rate constants as a function of temperature

We estimated the entropy and enthalpy of activation using the Eyring equation in the form

$$k_{\mathrm{d}} = \frac{k_{\mathrm{B}}T}{h}\exp\left(\frac{\Delta S^{\ddagger}}{R} - \frac{\Delta H^{\ddagger}}{RT}\right) \qquad \textit{(Eq. S7)}$$

which is convenient to fit with a linear function when modified as follows:

$$\mathrm{R}\ln\left(\frac{k_{\mathrm{d}}\cdot h}{k_{\mathrm{B}}\cdot T}\right) = -\Delta H^{\ddagger}\left(\frac{1}{T}\right) + \Delta S^{\ddagger} \qquad \textit{(Eq. S8)}$$

Supplementary Methods: ^{15}N chemical shifts of ligands and comparison of ESOTHERIC and phINEPT+ SOT performance

Table S2. ^{15}N Chemical shifts for the bound and free substrates ^{15}N-Py, ^{15}N-4AP, ^{15}N-NAM, ^{15}N-ACN, and $^{15}N_3$-MNZ. The third column is the ratio of the maximal polarizations obtained with ESOTHERIC and phINEPT+ at optimal conditions and 288 K for all substrates except $^{15}N_3$-MNZ (and at 267 K for $^{15}N_3$-MNZ).

Substrate \ ^{15}N Chemical shift	Bound substrate (ppm)	Free substrate (ppm)	$\frac{P^{max}_{ESOTHERIC}}{P^{max}_{phINEPT+}}$
[^{15}N]pyridine	256	300	1.08
4-amino[^{15}N]pyridine	215	256	1.38
[^{15}N]nicotinamide	256	302	1.3
[^{15}N]acetonitrile	179.5	240	1.28
[$^{15}N_3$]metronidazole	203.1	247.3	1.14

Supplementary Discussion: Phase distortion in the ESOTHERIC SOT sequence

The spectra plotted in **Figure 3** of the manuscript show NMR line distortions. These distortions are coming from zero and higher-order quantum coherences and the fact that the specta are measured directly after ESOTHERIC or phINEPT+ SOT sequences without any filters. To confirm that, we compared SABRE-ESOTHERIC NMR spectra measured with pulsed field gradient (PFG, filter of quantum coherences above one) during τ_e interval, with pulse field gradient and a zero-quantum (ZQ) filter and without any of this (**Figure S1**). Both PFG and ZQ filters contribute to the improvement of the line shape. The kinetic measurements during the study used only PFG. As exemplified here, the contribution of the ZQ filter changes the amplitude only by about 6% but, at the same time, does not allow for the measurement of exchange at times below 10 ms due to the ZQ filter.

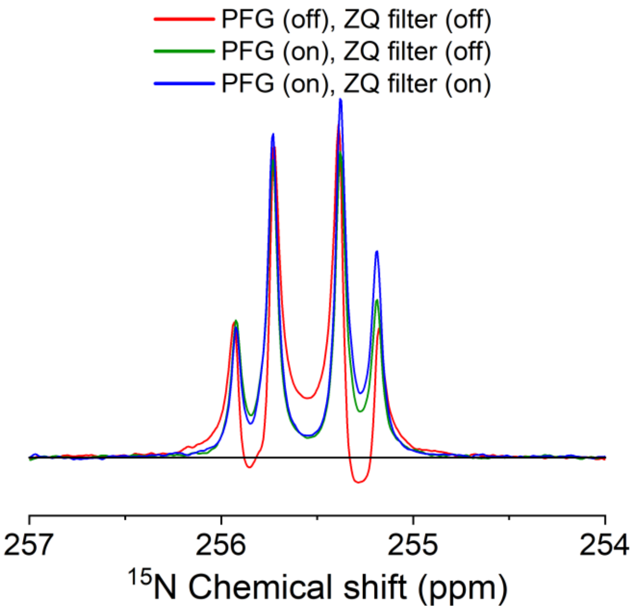

Figure S1. ^{15}N spectra of equatorial pyridine hyperpolarized with SABRE-ESOTHERIC. PFG and ZQ filters were added between the last two pulses (see **Figure 2C**): spectrum without any filters (red), spectrum with 2 ms of PFG (31% of SMSQ10.100, green), and spectrum with the same PFG and 10 ms of ZQ filter (blue). Integrals were measured to check the difference between these measurements, which showed an 8% higher signal using only PFG and 14% higher signal when using both PFG and ZQ filters.

Supplementary Discussion: Exchange rate constants measured with SABRE-ESOTHERIC

Figures S2-S6 present all measured SABRE-ESOTHERIC kinetics and biexponential fittings. **Table S3** provides the resulting fitting parameters.

Table S3. Modeled substrate dissociation rate constants using SABRE-ESOTHERIC data and two exchange models ($C_SS_2 \leftrightarrow C_SS+S$ and $C_SS_2 \leftrightarrow S_2$)[10] for ^{15}N-Py, n.a. Py,^{15}N-4AP, ^{15}N-NAM, ^{15}N-ACN, and $^{15}N_3$-MNZ. Dissociation rate constants measured in the previous work using SEXSY experiments[10] are presented for comparison (marked with asterisks). Error intervals are standard deviation values for the given variables obtained from fitting using MATLAB nonlinear regression function "nlinfit". Weighted mean k_d values were calculated using Eq. S6. Note that for $^{15}N_3$-MNZ we considered total dissociation flux, which fit well only using biexponential fitting; therefore, other fields are left empty.

Substrate (pH after bubbling)	Calibrated *T* (K)	$K = \frac{k_d}{k'_a} = \frac{[S]}{[C_SS_2]}$	*Model* $C_SS_2 \leftrightarrow C_SS+S$	*Model* $C_SS_2 \leftrightarrow S_2$	*Model* $C_SS_2 \leftrightarrow S_2$: Eigenvalues analysis (biexponential fitting)			Weighted mean k_d (s^{-1})
			k_d (s^{-1})	k_d (s^{-1})	k (s^{-1})	R (s^{-1})	k_d (s^{-1}) from k	
^{15}N-Py	279.3	7.22<br>7.46*	4.0 ± 0.4<br>1.9 ± 0.1*	4.1 ± 0.5<br>1.7 ± 0.1*	1.71 ± 0.05<br>1.55 ± 0.06*	0.061 ± 0.001<br>0.15 ± 0.02*	2.7 ± 0.1<br>2.4 ± 0.1*	3.6 ± 0.3<br>1.8 ± 0.1*
	282.5	9.4<br>8.61*	6.1 ± 0.5<br>3.4 ± 0.1*	6.4 ± 0.8<br>3.0 ± 0.1*	2.4 ± 0.1<br>2.6 ± 0.2*	0.061 ± 0.001<br>0.10 ± 0.06*	4.0 ± 0.2<br>4.2 ± 0.3*	5.5 ± 0.5<br>3.1 ± 0.2*
	287.9	8.15<br>9.72*	9.8 ± 0.8<br>7.6 ± 0.2*	11 ± 1<br>6.4 ± 0.3*	4.9 ± 0.3<br>4.6 ± 0.2*	0.055 ± 0.002<br>0.08 ± 0.03*	7.9 ± 0.4<br>7.7 ± 0.4	9.6 ± 0.7<br>6.3 ± 0.3
	293.4	6.7<br>15.82*	17 ± 3<br>14.9 ± 0.9*	21 ± 3<br>10.5 ± 0.8*	8.5 ± 0.4<br>8.0 ± 0.6*	0.054 ± 0.004<br>0.18 ± 0.06*	13.2 ± 0.7<br>14 ± 1*	17.1 ± 1.9<br>11.4 ± 0.9*
n.a. Py	279.3	49	3.0 ± 0.1	3.0 ± 0.1	1.52 ± 0.08	0.016 ± 0.004	2.9 ± 0.1	3.0 ± 0.1
	282.5	49	4.7 ± 0.1	4.7 ± 0.2	2.3 ± 0.1	0.018 ± 0.003	4.7 ± 0.2	4.7 ± 0.2
	287.9	49	8.3 ± 0.3	8.3 ± 0.3	4.1 ± 0.2	0.022 ± 0.003	7.7 ± 0.4	8.1 ± 0.3
	293.4	49	15.0 ± 0.6	15.5 ± 0.7	8.05 ± 0.08	0.016 ± 0.003	17.5 ± 0.9	16.0 ± 0.7
^{15}N-4AP	279.3	19.8<br>21.86*	1.6 ± 0.1<br>0.80 ± 0.04*	1.6 ± 0.1<br>0.90 ± 0.04*	0.71 ± 0.02<br>0.82 ± 0.01*	0.031 ± 0.001<br>0.08 ± 0.01*	1.31 ± 0.04<br>1.51 ± 0.03*	1.50 ± 0.08<br>0.97 ± 0.04*
	282.5	19.3<br>20.83*	2.1 ± 0.1<br>1.4 ± 0.1*	2.2 ± 0.1<br>1.7 ± 0.1*	1.12 ± 0.02<br>1.09 ± 0.03*	0.028 ± 0.001<br>0.09 ± 0.01*	1.99 ± 0.03<br>1.99 ± 0.05*	2.11 ± 0.06<br>1.52 ± 0.09*
	287.9	20.1<br>22.48*	3.8 ± 0.1<br>3.6 ± 0.1*	3.8 ± 0.1<br>3.6 ± 0.1*	2.02 ± 0.03<br>2.01 ± 0.09*	0.033 ± 0.001<br>0.09 ± 0.01*	3.68 ± 0.06<br>3.7 ± 0.2*	3.8 ± 0.1<br>3.2 ± 0.1*
	293.4	21.3<br>23.85*	6.7 ± 0.3<br>5.5 ± 0.6*	6.7 ± 0.3<br>8 ± 3*	3.47 ± 0.08<br>2.9 ± 0.5*	0.034 ± 0.006<br>0.10 ± 0.02*	6.4 ± 0.1<br>5.4 ± 0.8*	6.6 ± 0.3<br>6 ± 1*
^{15}N-NAM	279.3	11.7<br>23.48*	2.4 ± 0.3<br>1.5 ± 0.1*	2.3 ± 0.4<br>1.3 ± 0.1*	0.96 ± 0.07<br>1.20 ± 0.06*	0.08 ± 0.01<br>0.16 ± 0.01*	1.6 ± 0.1<br>2.2 ± 0.1*	2.1 ± 0.3<br>1.40 ± 0.09*
	282.5	14.3<br>24.25*	3.0 ± 0.4<br>2.1 ± 0.1*	3.8 ± 0.6<br>2.1 ± 0.1*	1.4 ± 0.1<br>1.62 ± 0.09*	0.08 ± 0.01<br>0.15 ± 0.01*	2.4 ± 0.2<br>3.0 ± 0.2*	3.1 ± 0.4<br>2.0 ± 0.1*
	287.9	10.56<br>24.92*	5.1 ± 0.2<br>3.5 ± 0.2*	5.1 ± 0.2<br>5.8 ± 0.6*	2.72 ± 0.07<br>1.73 ± 0.09*	0.078 ± 0.005<br>0.16 ± 0.01*	4.6± 0.1<br>3.2 ± 0.2*	4.9 ± 0.2<br>3.7 ± 0.3*
	293.4	12.24<br>26.37*	10.0 ± 0.3<br>6.3 ± 0.4*	10.0 ± 0.3<br>9.9 ± 0.8*	4.63 ± 0.07<br>4.1 ± 0.3*	0.085 ± 0.006<br>0.12 ± 0.01*	8.0 ± 0.1<br>7.6 ± 0.6*	9.3 ± 0.2<br>7.4 ± 0.6*
^{15}N-ACN	260.3	7.68	2.0 ± 0.1	2.0 ± 0.1	0.98 ± 0.04	0.042 ± 0.001	1.55 ± 0.06	1.9 ± 0.1
	265.2	7.68	3.5 ± 0.2	3.5 ± 0.2	1.70 ± 0.05	0.036 ± 0.002	2.68 ± 0.07	3.2 ± 0.2
	268.4	7.68	5.4 ± 0.2	5.4 ± 0.2	2.6 ± 0.1	0.034 ± 0.001	4.1 ± 0.2	5.0 ± 0.2
	272	7.68	8.4 ± 0.4	8.4 ± 0.4	3.9 ± 0.2	0.037 ± 0.001	6.1 ± 0.3	7.7 ± 0.4

	279.3	14.6	16.5 ± 0.4	16.5 ± 0.4	7.8 ± 0.1	0.023 ± 0.001	13.7 ± 0.2	15.6 ± 0.3
	282.5	14.6	22.8 ± 0.5	23.8 ± 0.4	11.5 ± 0.2	0.019 ± 0.001	20.3 ± 0.3	22.3 ± 0.4
	287.9	14.6	45.5 ± 0.9	45.6 ± 0.9	22.3 ± 0.5	0.028 ± 0.002	39.4 ± 0.8	43.4 ± 0.9
	293.4	14.6	58 ± 2	60 ± 2	45 ± 2	0.047 ± 0.004	80 ± 3	66 ± 2
	257.6	6.64			0.67 ± 0.04	0.08 ± 0.01	1.03 ± 0.05	
	260.3	6.64			0.94 ± 0.02	0.067 ± 0.003	1.45 ± 0.03	
	265.2	6.64			1.40 ± 0.02	0.064 ± 0.002	2.16 ± 0.03	
	268.4	6.64			1.99 ± 0.03	0.062 ± 0.001	3.06 ± 0.04	
$^{15}N_3$-MNZ	272	6.64			2.82 ± 0.06	0.061 ± 0.001	4.3 ± 0.1	
	273.84	6.64			3.6 ± 0.1	0.063 ± 0.002	5.6 ± 0.2	
	276	6.64			4.69 ± 0.07	0.055 ± 0.001	7.2 ± 0.1	
	279.3	6.64			6.9 ± 0.1	0.051 ± 0.001	10.7 ± 0.2	
	282.5	6.64			9.8 ± 0.2	0.048 ± 0.001	15.1 ± 0.3	
	286.8	6.64			15.5 ± 0.4	0.041 ± 0.001	23.8 ± 0.6	

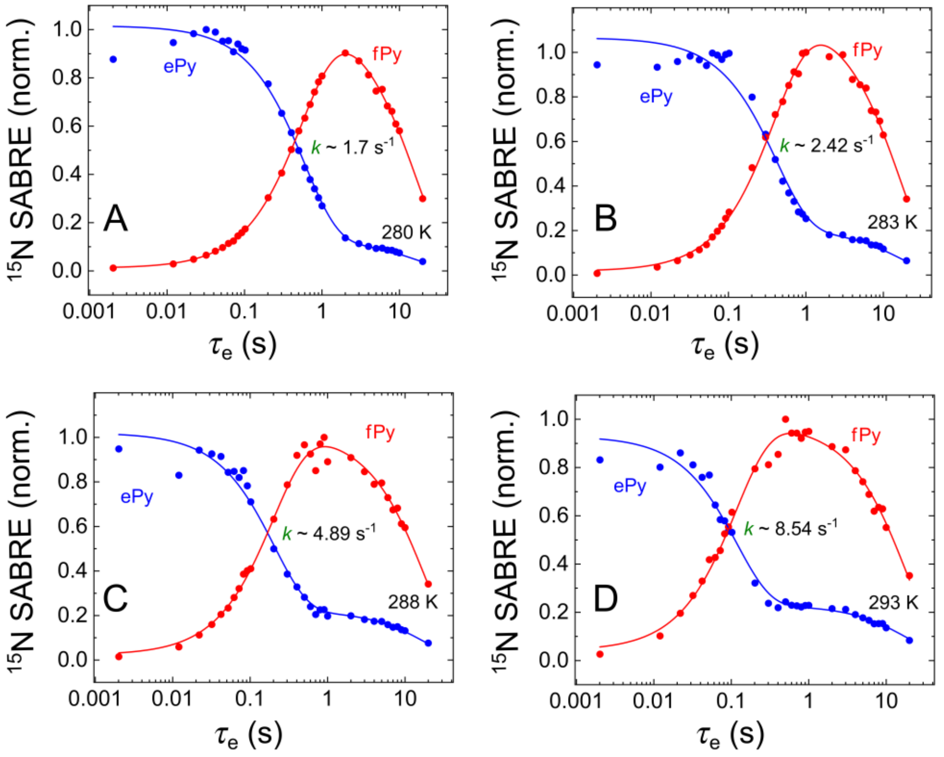

Figure S2. Dependences of the SABRE ESOTHERIC-enhanced ^{15}N NMR signals of ^{15}N-ePy (blue) and ^{15}N-fPy (red) on the inter-pulse delay τ_e with τ_1 = 7 ms and τ_2 = 6 ms at the nominal temperatures of 280 K (A), 283 K (B), 288 K (C), and 293 K (D). The curves are fitted using biexponential global fit. Estimated exchange rate constants are given in **Table S3**.

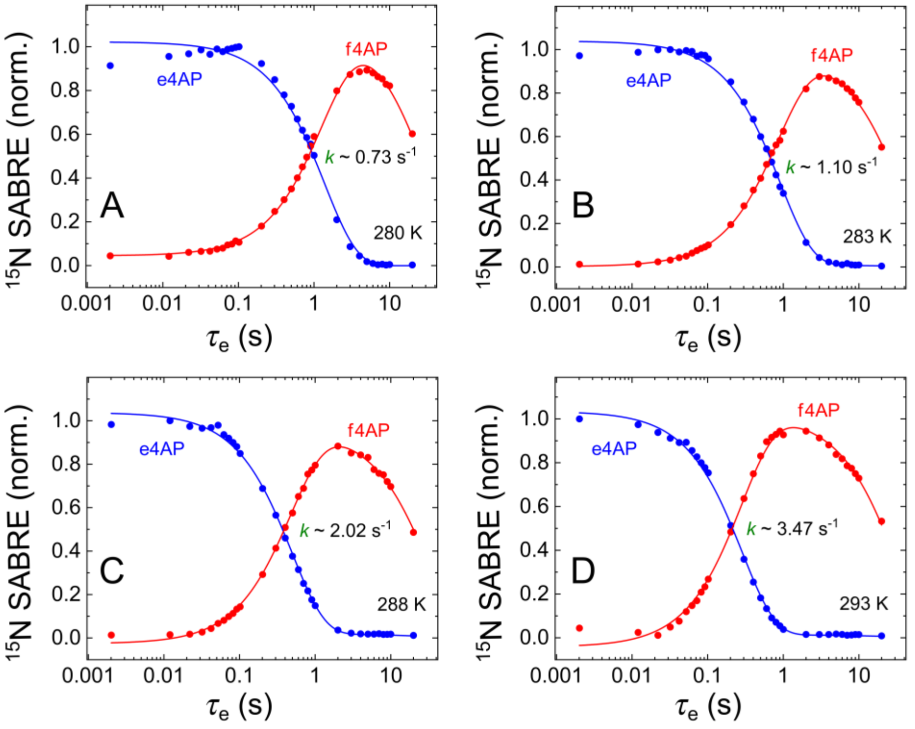

Figure S3. Dependences of the SABRE ESOTHERIC-enhanced ^{15}N NMR signals of ^{15}N-e4AP (blue) and ^{15}N-f4AP (red) on the inter-pulse delay τ_e with τ_1 = 7 ms and τ_2 = 5 ms at the nominal temperatures of 280 K (A), 283 K (B), 288 K (C), and 293 K (D). The curves are fitted using biexponential global fit. Estimated exchange rate constants are given in **Table S3**.

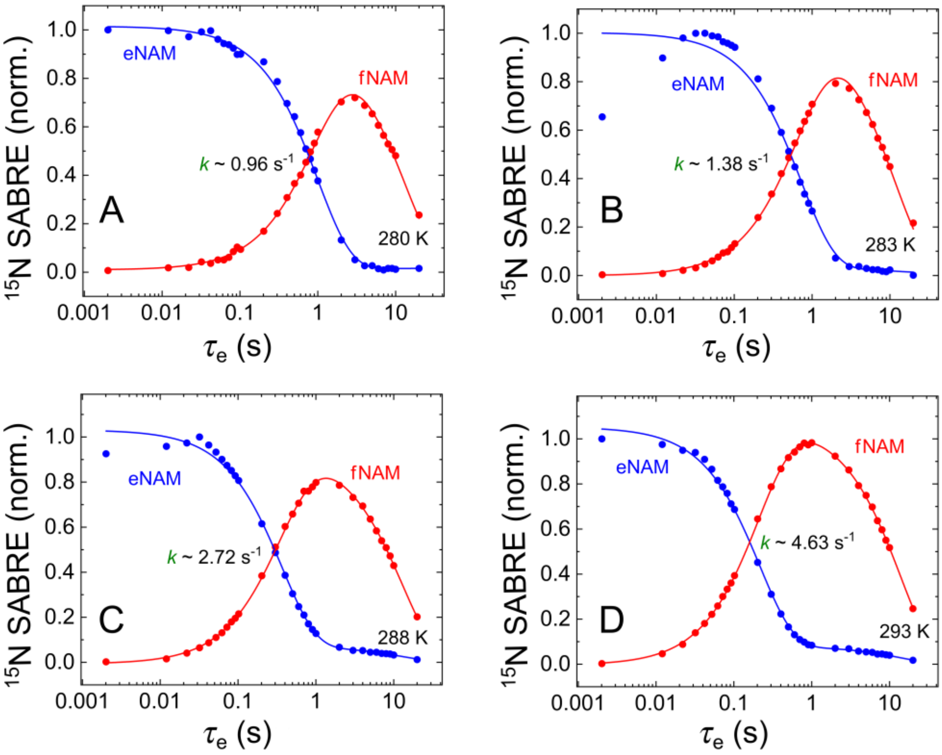

Figure S4. Dependences of the SABRE ESOTHERIC-enhanced ^{15}N NMR signals of ^{15}N-eNAM (blue) and ^{15}N-fNAM (red) on the inter-pulse delay τ_e with τ_1 = 7 ms and τ_2 = 5 ms at the nominal temperatures of 280 K (A), 283 K (B), 288 K (C), and 293 K (D). The curves are fitted using biexponential global fit. Estimated exchange rate constants are given in **Table S3**.

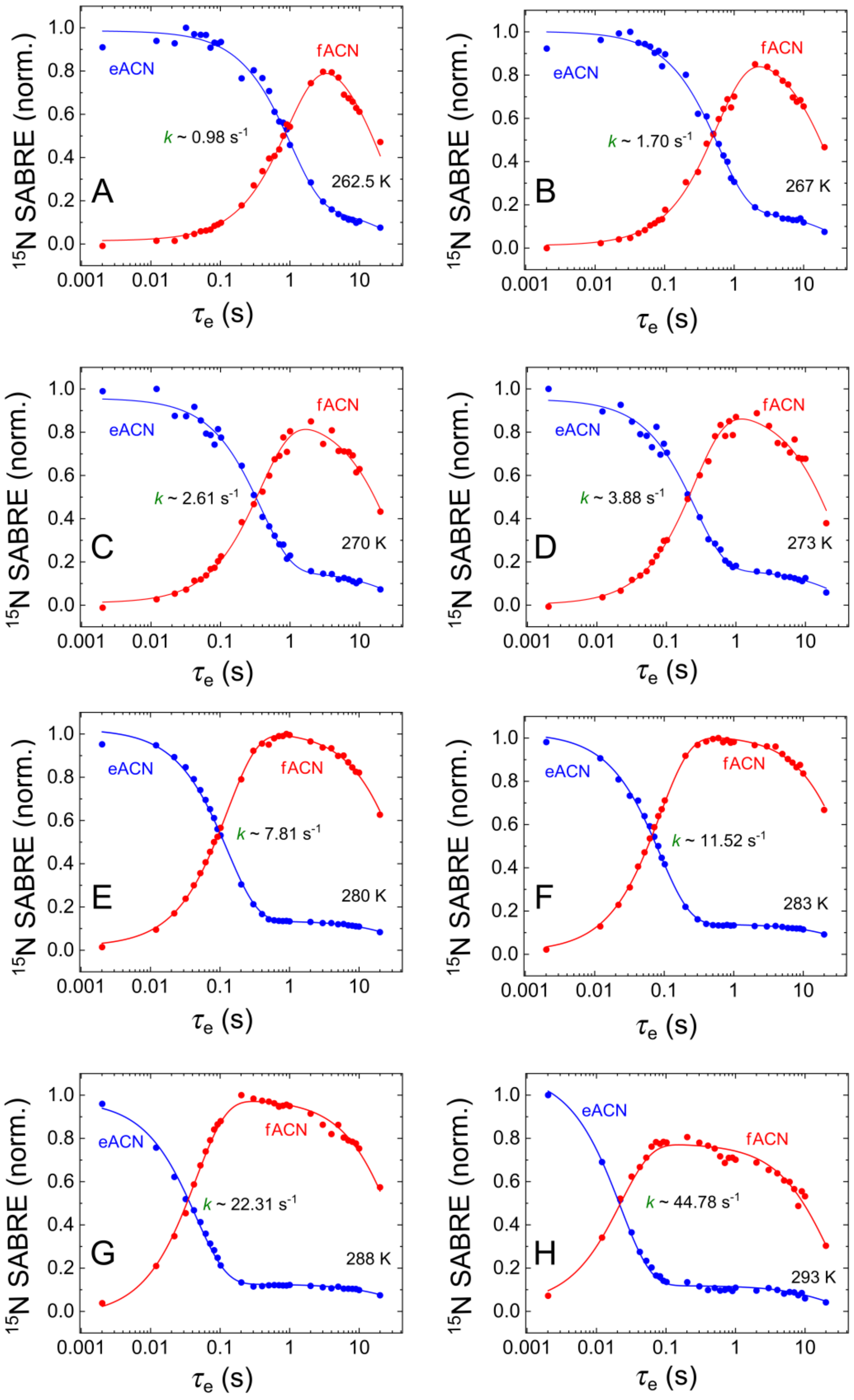

Figure S5. Dependences of the SABRE ESOTHERIC-enhanced ^{15}N NMR signals of ^{15}N-eACN (blue) and ^{15}N-fACN (red) on the inter-pulse delay τ_e with τ_1 = 5 ms and τ_2 = 4 ms at the nominal temperatures of 262.5 K (A), 267 K (B), 270 K (C), 273 K (D), 280 K (E), 283 K (F), 288 K (G), and 293 K (H). The curves are fitted using biexponential global fit. Estimated exchange rate constants are given in **Table S3**.

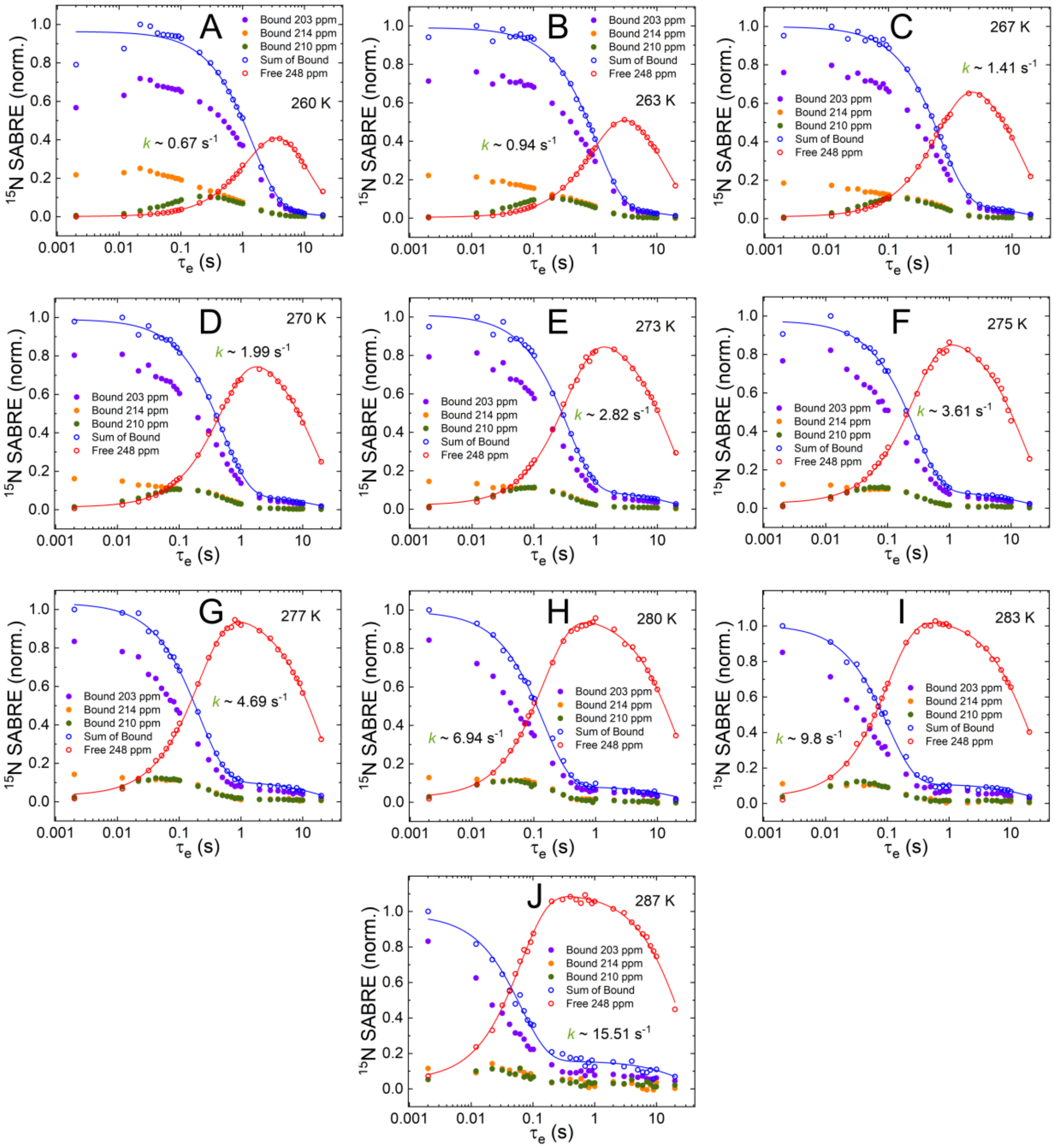

Figure S6. Dependences of the SABRE ESOTHERIC-enhanced ^{15}N NMR signals of $^{15}N_3$-eMNZ (blue) and $^{15}N_3$-fMNZ (red) on the inter-pulse delay τ_e with τ_1 = 6 ms and τ_2 = 8 ms at the nominal temperatures of 260 K (A), 263 K (B), 267 K (C), 270 K (D), 273 K (E), 275 K (F), 277 K (G), 280 K (H), 283 K (I), and 287 K (J). The integrals of three signals of $^{15}N_3$-eMNZ in different complexes (see **Figure S8** and **Figure S10**) with chemical shifts of 203 ppm (purple), 210 ppm (green), and 214 ppm (yellow) are presented as well as the sum of these integrals (blue). The curves are fitted using biexponential global fit. Estimated exchange rate constants are given in **Table S3**.

Supplementary Discussion: Comparison of exchange rate constants with literature data

Here we compare the exchange rate constants measured for pyridine using SABRE-ESOTHERIC with the data from previous studies where SABRE-INEPT[11] or SEXSY[10] approaches were employed. The corresponding values are presented in **Table S4A–S4C** (eigenvalues analysis was used for data fitting). Ideally, the choice of method should not affect the obtained values; however, in reality, they can be different due to systematic or random errors of particular approaches. In the previous SABRE-INEPT study,[11] k_d values were originally calculated as $k_\mathrm{d} = \frac{k}{1+\frac{[\mathrm{C_S S_2}]}{[\mathrm{S}]}}$ because single-site exchange model was used. Thus, we corrected these values by using our $\mathrm{C_S S_2} \leftrightarrow \mathrm{S_2}$ model which gives $k_\mathrm{d} = \frac{k}{0.5+\frac{[\mathrm{C_S S_2}]}{[\mathrm{S}]}}$.

Table S4A. Exchange rate constants for pyridine obtained using SABRE-INEPT approach in Ref. [11]. Published values give $\Delta H^{0\ddagger}$ = 78 ± 2 kJ/mol, $\Delta S^{0\ddagger}$ = 41 ± 8 J/(mol·K). Recalculated values give $\Delta H^{0\ddagger}$ = 79 ± 2 kJ/mol, $\Delta S^{0\ddagger}$ = 48 ± 8 J/(mol·K).

T (K)	k_d (s^{-1}) published	k_d (s^{-1}) recalculated according to $\mathrm{C_S S_2} \leftrightarrow \mathrm{S_2}$ model (Eq. 2)
273.15	0.9 ± 0.1	1.6 ± 0.2
286.65	3.9 ± 0.1	7.2 ± 0.2
291.15	6.9 ± 0.2	12.6 ± 0.3
298.15	16.4 ± 0.9	30 ± 1
301.15	21 ± 3	38 ± 5

Table S4B. Exchange rate constants for pyridine obtained using SEXSY approach in Ref. [10]. –These values give $\Delta H^{0\ddagger}$ = 91 ± 2 kJ/mol, $\Delta S^{0\ddagger}$ = 90 ± 8 J/(mol·K).

T (K)	k_d (s^{-1})
280	2.4 ± 0.1
283	4.2 ± 0.3
288	7.7 ± 0.4
293	14 ± 1
298	29 ± 2

Table S4C. Exchange rate constants for pyridine were obtained using SABRE-ESOTHERIC approach in this work. -These values give $\Delta H^{0\ddagger}$ = 83 ± 2 kJ/mol, $\Delta S^{0\ddagger}$ = 61 ± 7 J/(mol·K).

T (K)	k_d (s^{-1})
280	2.7 ± 0.1
283	4.0 ± 0.2
288	7.9 ± 0.4
293	13.2 ± 0.7

Supplementary Discussion: Enthalpies and entropies of activation

Eyring plots used for estimation of enthalpies and entropies of activation $\Delta H^{\ddagger}$ and $\Delta S^{\ddagger}$ (from ligand dissociation rate constants k_d presented in **Table S3**) are shown in **Figure S7**.

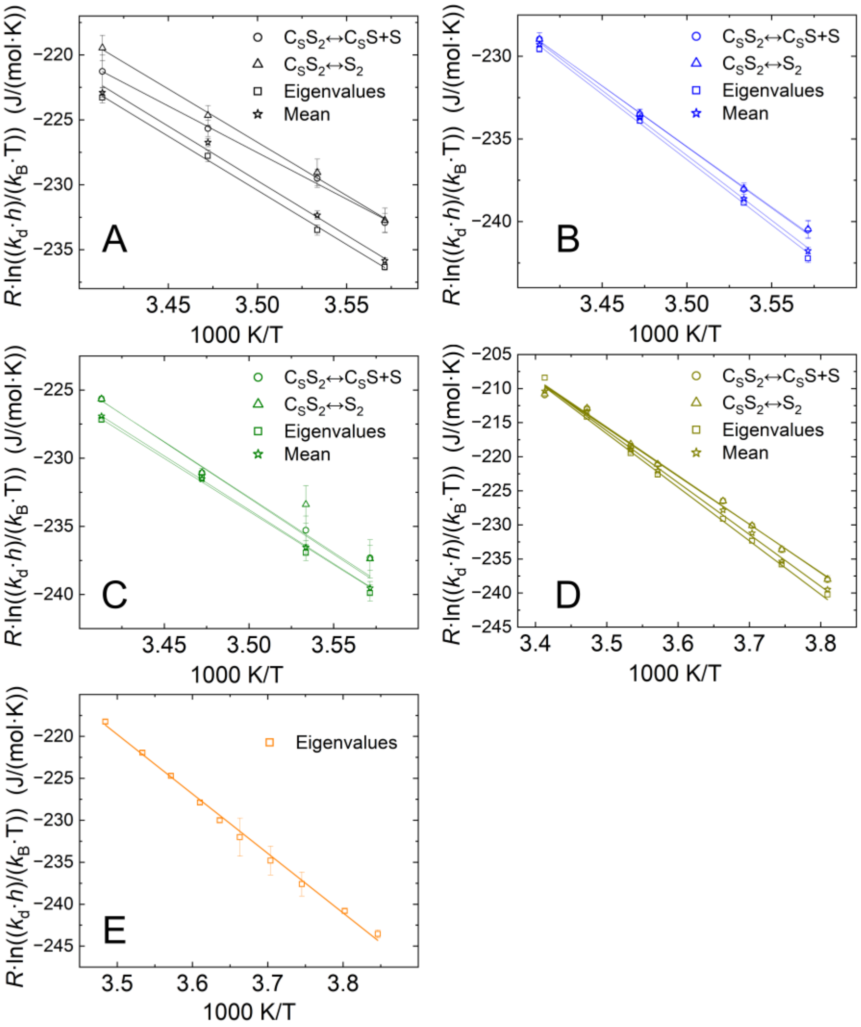

Figure S7. **Eyring plots for (A) [^{15}N]pyridine, (B) 4-amino[^{15}N]pyridine, (C) [^{15}N]nicotinamide, (D) [^{15}N]acetonitrile, and (E) [$^{15}N_3$]metronidazole**. Dissociation rate constants k_d were obtained by fitting SABRE-ESOTHERIC kinetics with the models $C_SS_2 \leftrightarrow C_SS+S$ (circles), $C_SS_2 \leftrightarrow S_2$ (triangles), eigenvalues analysis (squares), and weighted mean values across the three models (stars). Here, these values are displayed in Eyring coordinates.

In **Table S5**, the enthalpies $\Delta H^{\ddagger}$ and entropies $\Delta S^{\ddagger}$ of activation for all substrates obtained from dissociation rate constants k_d (**Table S3**) measured in this work using SABRE-ESOTHERIC approach are presented. Additionally, we compared our results to the enthalpies $\Delta H^{\ddagger}$ and entropies $\Delta S^{\ddagger}$ of activation for the substrates measured in the previous study[10] using SEXSY approach (pyridine, 4-aminopyridine, and nicotinamide). The measurements conducted in this work appear to be more robust.

Table S5. Enthalpies $\Delta H^{\ddagger}$ and entropies $\Delta S^{\ddagger}$ of activation for all substrates. $\Delta H^{\ddagger}$ and $\Delta S^{\ddagger}$ were obtained from dissociation rate constants k_d (**Table S3**) and from the previous work[10] (marked with asterisks). Measurements and fitting using SABRE-ESOTHERIC appear more robust than the SEXSY approach used in the previous study.[10]

Substrate	From mean k_d values		Model $C_SS_2 \leftrightarrow C_SS+S$		Model $C_SS_2 \leftrightarrow S_2$		Eigenvalues analysis: biexponential fitting	
	$\Delta H^{\ddagger}$ (kJ/mol)	$\Delta S^{\ddagger}$ (J/(mol·K))	$\Delta H^{\ddagger}$ (kJ/mol)	$\Delta S^{\ddagger}$ (J/(mol·K))	$\Delta H^{\ddagger}$ (kJ/mol)	$\Delta S^{\ddagger}$ (J/(mol·K))	$\Delta H^{\ddagger}$ (kJ/mol)	$\Delta S^{\ddagger}$ (J/(mol·K))
^{15}N-Py	84 ± 4<br>104 ± 5*	64 ± 16<br>133 ± 17*	72 ± 4<br>109 ± 3*	24 ± 14<br>150 ± 11*	82 ± 3<br>101 ± 6*	61 ± 11<br>122 ± 21*	83 ± 2<br>91 ± 2*	61 ± 7<br>90 ± 7*
^{15}N-4AP	79 ± 1<br>95 ± 1*	39 ± 5<br>98 ± 2*	74 ± 2<br>101 ± 16*	23 ± 6<br>115 ± 56*	73 ± 2<br>112 ± 5*	21 ± 6<br>115 ± 18*	79 ± 2<br>71 ± 3*	41 ± 8<br>12 ± 11*
^{15}N-NAM	79 ± 1<br>81 ± 2*	42 ± 3<br>48 ± 6*	81 ± 7<br>66 ± 3*	52 ± 22<br>4 ± 13*	82 ± 9<br>107 ± 6*	55 ± 30<br>141 ± 21*	78 ± 3<br>49 ± 12*	39 ± 11<br>59 ± 43*
^{15}N-ACN	76 ± 2	71 ± 3	71 ± 3	32 ± 11	71 ± 3	33 ± 11	79 ± 2	59 ± 7
$^{15}N_3$-MNZ							71 ± 1	29 ± 5

Supplementary Discussion: Analysis of SABRE complexes with metronidazole

When ESOTHERIC or phINEPT+ SOTs were applied to [$^{15}N_3$]MNZ, we observed multiple ^{15}N peaks of ^{15}N-3 sites of $^{15}N_3$-MNZ: one for the free substrate at 247.3 ppm, one for the equatorial-bound substrate in the [Ir(IMes)(H)$_2$(MNZ)$_3$] complex at 203.1 ppm and several additional peaks at 196, 205, 206.5, and 214 ppm (**Figure S8**). The latter peaks likely belong to various Ir complexes with MNZ and additional ligands: chloride or solvent[12,13] (**Figure S9A**).

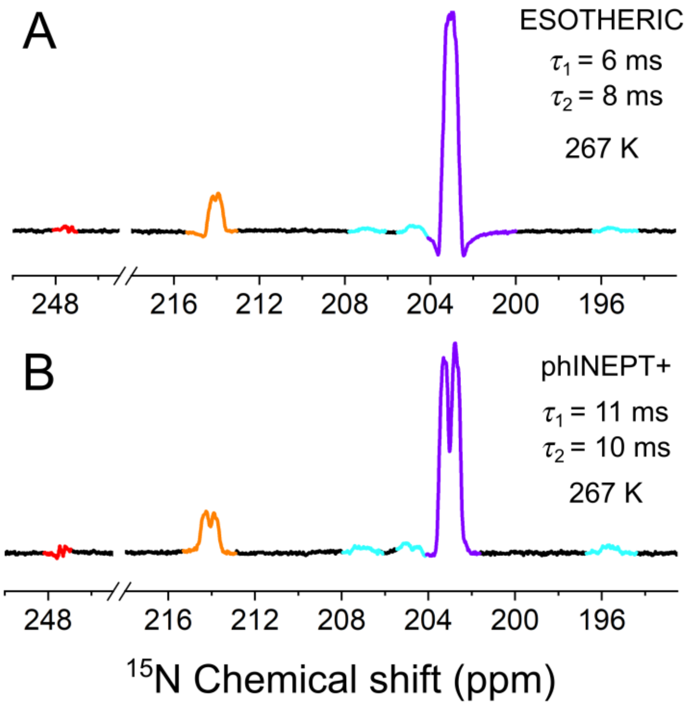

Figure S8. ^{15}N ESOTHERIC (A) and phINEPT+ (B) NMR spectra of [$^{15}N_3$]metronidazole. ^{15}N symmetrically bound metronidazole resonances at 203.68 ppm (purple), at 214 ppm (orange), and the ^{15}N free metronidazole resonance at 247.3 ppm (red). The peaks at 196, 205, and 206.5 ppm correspond to different confirmations of MNZ-Ir complexes (sky blue).

To assess the efficiency of polarization transfer to different Ir-MNZ complexes, we measured ^{1}H PASADENA spectra at various temperatures (**Figure S9C**). There are three major iridium complexes, which can be assigned as [Ir(IMes)(H)$_2$(MNZ)$_3$] (**1**), [Ir(IMes)(H)$_2$(MNZ)$_2$Cl] (**2**), and [Ir(IMes)(H)$_2$(MNZ)$_2$(Solv)] (**3**).[12] Increasing pH$_2$ pressure enhanced the polarization proportionally, and several minor hydride species, such as complex (**3**), became visible even better (**Figure S9B**). We found that at 8.3 bar, the highest polarization for complex (**1**) reached 277 K (**Figure S9D**), while for (**2**) the strongest polarization was observed at 260 K. The sample composition was [Ir] = 4 mM, [MNZ] = 80 mM in 400 µL of methanol-d_4.

Also, the performance of phINEPT+ and ESOTHERIC SOT sequences for polarization transfer in various $^{15}N_3$-MNZ-Ir complexes was experimentally evaluated (**Figure S10**). We found that the τ_1 and τ_2 delays optimized for the signal at 203.1 ppm (corresponding to complex (**1**)) are suboptimal for the polarization transfer in other complexes.

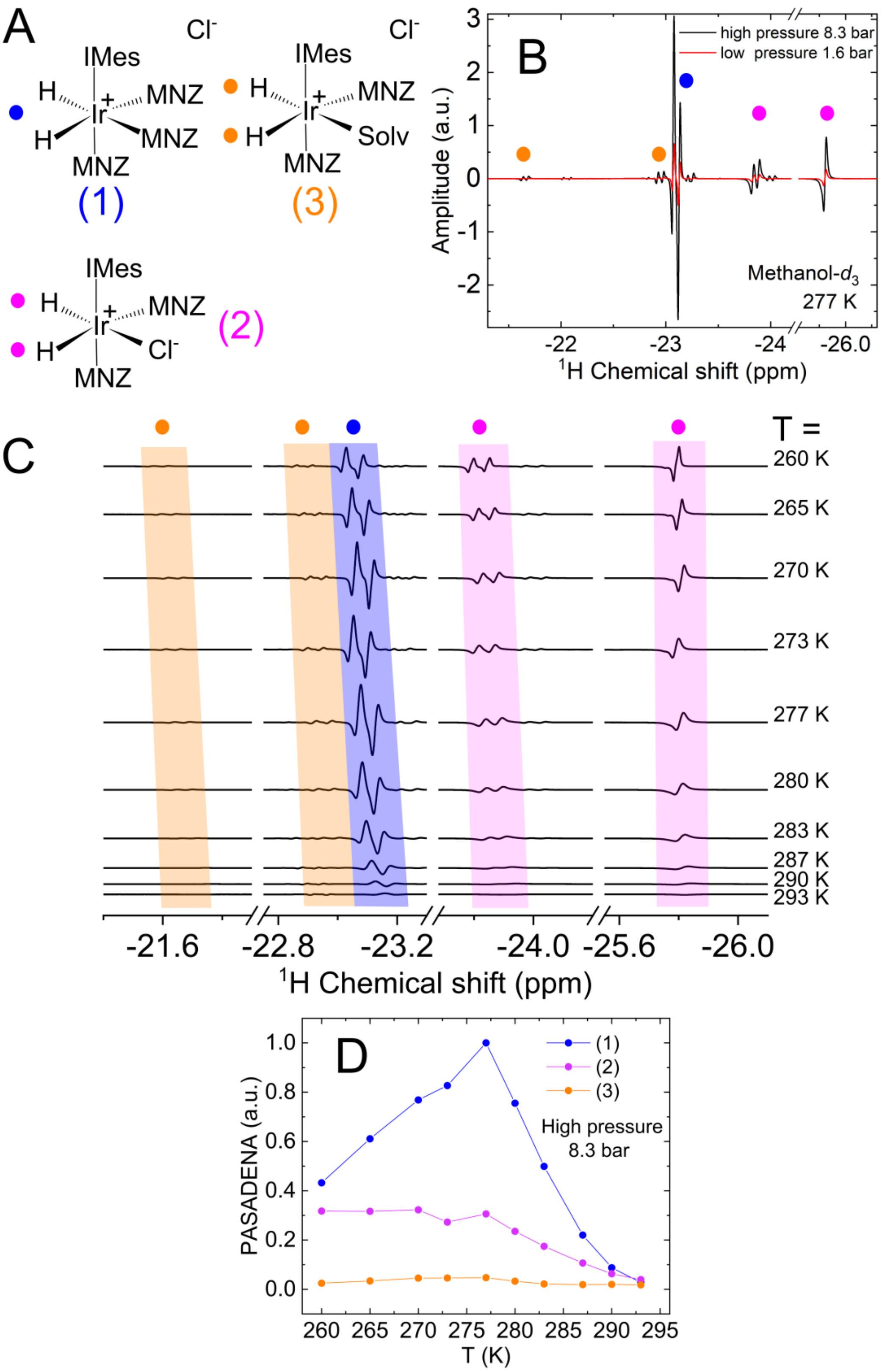

Figure S9. PASADENA ^{1}H NMR spectra of SABRE complexes with MNZ. (A) The structures of the three SABRE complexes with MNZ. (B) ^{1}H PASADENA spectra at high pressure (8.3 bar, black) and low pressure (1.6 bar, red). (C) ^{1}H PASADENA spectra as a function of temperature between 260 and 293 K recorded at 8.3 bar. ^{1}H PASADENA magnitude integrals of the three primary complexes **(1)-(3)** as a function of temperature at 8. 3 bar (D).

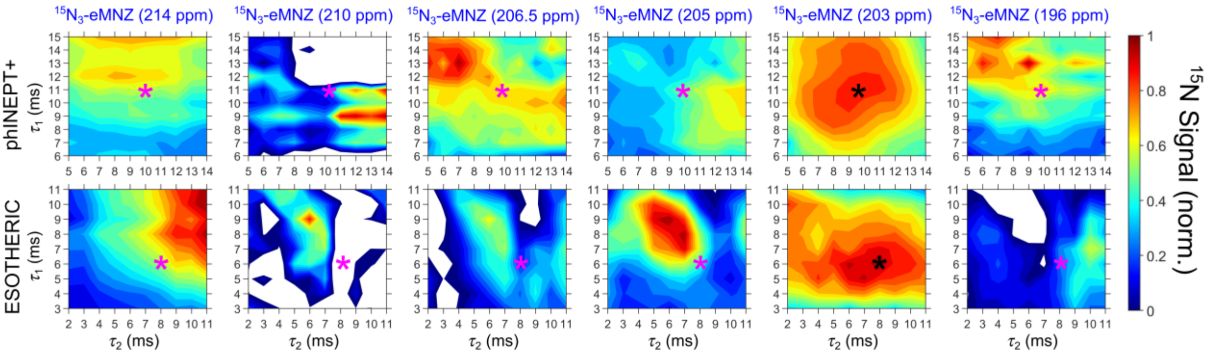

Figure S10. Performance of ESOTHERIC and phINEPT+ SOTs for various ^{15}N NMR signals of Ir-bound $^{15}N_3$-MNZ. Experimentally measured ^{15}N polarization of [$^{15}N_3$]metronidazole ($^{15}N_3$-MNZ) as a function of τ_1 and τ_2 delays of phINEPT+ (top) and ESOTHERIC (bottom) SOT sequences at 267 K and 9.4 T. The signals of the ^{15}N-3 site at 196, 203, 205, 206.5, 210, and 214 ppm correspond to different MNZ-Ir complexes. The asterisks represent the τ_1 and τ_2 delays that were used to measure the ^{15}N ESOTHERIC and phINEPT+ NMR spectra of [$^{15}N_3$]metronidazole presented in **Figure S8**.

Supplementary Discussion: N.a. pyridine exchange

We performed SABRE-ESOTHERIC measurements using pyridine with the natural abundance of ^{15}N nuclei. The sample was prepared by mixing 400 mM of pyridine with 4 mM of [Ir-d_{22}] in 0.6 mL of methanol-*d*$_4$. First, we experimentally estimated the optimum parameters for phINEPT+ and ESOTHERIC for polarization transfer from pH_2-derived hydrides to the equatorially bound pyridine on the SABRE catalyst. We varied the two time intervals τ_1 and τ_2 (**Figure 7A**) and measured the ^{15}N signal of the bound ^{15}N natural abundance pyridine at 280 K. The SABRE-ESOTHERIC kinetics for n.a. pyridine are presented in **Figure S11**.

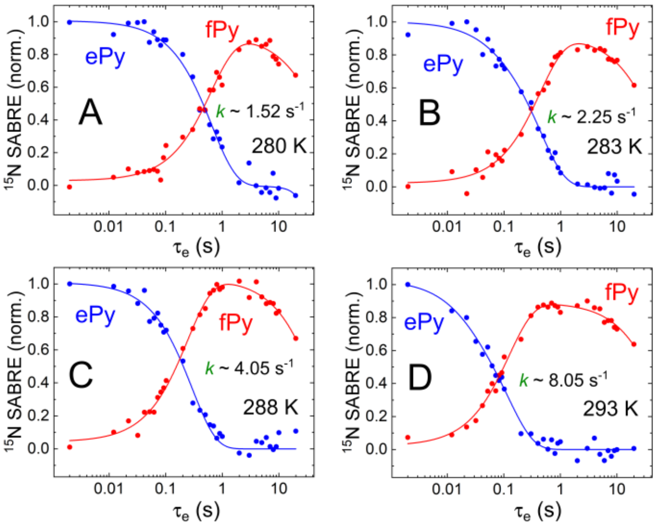

Figure S11. Dependences of the SABRE-ESOTHERIC-enhanced ^{15}N NMR signals of n.a. ePy (blue) and n.a. fPy (red) on the inter-pulse delay τ_e with τ_1 = 9 ms and τ_2 = 6 ms at the nominal temperatures of 280 K (A), 283 K (B), 288 K (C), and 293 K (D)**.** The curves are fitted using biexponential global fit. Estimated exchange rate constants are given in **Table S3**.

Supplementary Discussion: Quantum chemical calculations

All quantum chemical calculations were performed with the ORCA 5.0 program package.[14] Geometries of $[Ir(H)_2(IMes)(S)_3]^+$, $[Ir(H)_2(IMes)(S)_2]^+$ and S (S = Py, 4AP, NAM, ACN, or MNZ) were optimized at the B3LYP-D4/def2-TZVP level of theory[15–18]. Harmonic vibrational frequency analyses confirmed the optimized structures as true minima. At the optimized geometries, single-point energies were calculated at the DLPNO-CCSD(T)/def2-TZVP level of theory[19,20]. The CPCM model was used to account for solvation effects in both geometry optimizations and single-point calculations.[21]

While great care has been taken to use well-established DFT functionals, state-of-the-art corrections for dispersion and solvation, and sufficiently large basis sets and grids, the overestimation of dissociation energies is very likely due to the insufficient description of the solvent stabilizing the dissociation products, or missing effects of similar origin.

To identify the transition state of ligand dissociation in $[Ir(H)_2(IMes)(Py)_3]^+$, a relaxed potential energy surface (PES) scan was conducted at the B3LYP-D4/def2-SVP level of theory. The Ir–N distance was scanned from 2.3 Å to 5 Å with an increment step of 0.1 Å, giving a total of 28 steps. The dissociation of the pyridine ligand has a pronounced energy barrier (**Figure S12**). Hence, the experimentally observed "activation energy" represents the energy required for the ligand to diffuse away from the Ir complex, rather than the energy needed to overcome a distinct barrier. All calculated energies are summarized in **Table S6**.

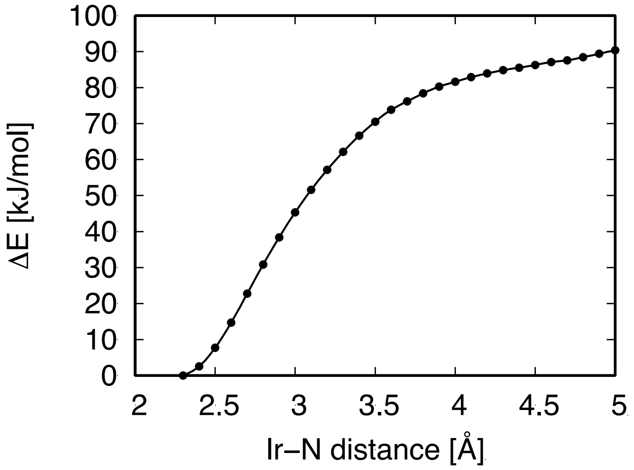

Figure S12. The obtained PES of ligand dissociation in $[Ir(H)_2(IMes)(Py)_3]^+$.

Table S6. Summary of B3LYP-D4 energies with zero-point energy correction and CCSD(T) energies for $[Ir(H)_2(IMes)(S)_3]^+$, $[Ir(H)_2(IMes)(S)_2]^+$ and S (S = Py, 4AP, NAM, ACN, MNZ).

Substrate	Compound	E(DFT) (Hartree)	ΔE(DFT) (kJ/mol)	E(CCSD(T)) (Hartree)	ΔE(CCSD(T)) (kJ/mol)
Py	$[Ir(H)_2(IMes)(Py)_3]^+$	-1773.6626	103.9383	-1771.0543	123.5654
	$[Ir(H)_2(IMes)(Py)_2]^+$	-1525.4642		-1523.1806	
	Py	-248.1588		-247.8267	
4AP	$[Ir(H)_2(IMes)(4AP)_3]^+$	-1939.7220	98.8423	-1936.9116	120.8313
	$[Ir(H)_2(IMes)(4AP)_2]^+$	-1636.1748		-1633.7554	
	4AP	-303.5096		-303.1102	
NAM	$[Ir(H)_2(IMes)(NAM)_3]^+$	-2279.7264	106.2501	-2276.4594	125.1863
	$[Ir(H)_2(IMes)(NAM)_2]^+$	-1862.8397		-1860.1169	
	NAM	-416.8462		-416.2948	
ACN	$[Ir(H)_2(IMes)(ACN)_3]^+$	-1427.2491	72.9700	-1425.1285	89.0197

	$[Ir(H)_2(IMes)(ACN)_2]^+$	-1294.5224		-1292.5629	
	ACN	-132.6989		-132.5317	
MNZ	$[Ir(H)_2(IMes)(MNZ)_3]^+$	-2900.2430	82.0146	-2896.2835	107.3883
	$[Ir(H)_2(IMes)(MNZ)_2]^+$	-2276.5239		-2273.3383	
	MNZ	-623.6878		-622.9043	

Supplementary References:

(1) Rayner, P. J.; Burns, M. J.; Olaru, A. M.; Norcott, P.; Fekete, M.; Green, G. G. R.; Highton, L. A. R.; Mewis, R. E.; Duckett, S. B. Delivering Strong ^{1} H Nuclear Hyperpolarization Levels and Long Magnetic Lifetimes through Signal Amplification by Reversible Exchange. *Proc. Natl. Acad. Sci.* **2017**, *114* (16). https://doi.org/10.1073/pnas.1620457114.

(2) Chukanov, N. V.; Salnikov, O. G.; Trofimov, I. A.; Kabir, M. S. H.; Kovtunov, K. V.; Koptyug, I. V.; Chekmenev, E. Y. Synthesis and ^{15} N NMR Signal Amplification by Reversible Exchange of [^{15} N]Dalfampridine at Microtesla Magnetic Fields. *ChemPhysChem* **2021**, *22* (10), 960–967. https://doi.org/10.1002/cphc.202100109.

(3) Shchepin, R. V.; Barskiy, D. A.; Mikhaylov, D. M.; Chekmenev, E. Y. Efficient Synthesis of Nicotinamide-1- ^{15} N for Ultrafast NMR Hyperpolarization Using Parahydrogen. *Bioconjug. Chem.* **2016**, *27* (4), 878–882. https://doi.org/10.1021/acs.bioconjchem.6b00148.

(4) Peters, J. P.; Brahms, A.; Janicaud, V.; Anikeeva, M.; Peschke, E.; Ellermann, F.; Ferrari, A.; Hellmold, D.; Held-Feindt, J.; Kim, N.; Meiser, J.; Aden, K.; Herges, R.; Hövener, J.-B.; Pravdivtsev, A. N. Nitrogen-15 Dynamic Nuclear Polarization of Nicotinamide Derivatives in Biocompatible Solutions. *Sci. Adv.* **2023**, *9* (34), eadd3643. https://doi.org/10.1126/sciadv.add3643.

(5) Shchepin, R. V.; Birchall, J. R.; Chukanov, N. V.; Kovtunov, K. V.; Koptyug, I. V.; Theis, T.; Warren, W. S.; Gelovani, J. G.; Goodson, B. M.; Shokouhi, S.; Rosen, M. S.; Yen, Y.; Pham, W.; Chekmenev, E. Y. Hyperpolarizing Concentrated Metronidazole ^{15} NO $_2$ Group over Six Chemical Bonds with More than 15 % Polarization and a 20 Minute Lifetime. *Chem. – Eur. J.* **2019**, *25* (37), 8829–8836. https://doi.org/10.1002/chem.201901192.

(6) Hövener, J.-B.; Bär, S.; Leupold, J.; Jenne, K.; Leibfritz, D.; Hennig, J.; Duckett, S. B.; von Elverfeldt, D. A Continuous-Flow, High-Throughput, High-Pressure Parahydrogen Converter for Hyperpolarization in a Clinical Setting: A HIGH-THROUGHPUT PARAHYDROGEN CONVERTER FOR HYPERPOLARIZATION. *NMR Biomed.* **2013**, *26* (2), 124–131. https://doi.org/10.1002/nbm.2827.

(7) Ellermann, F.; Pravdivtsev, A.; Hövener, J.-B. Open-Source, Partially 3D-Printed, High-Pressure (50-Bar) Liquid-Nitrogen-Cooled Parahydrogen Generator. *Magn. Reson.* **2021**, *2* (1), 49–62. https://doi.org/10.5194/mr-2-49-2021.

(8) Pravdivtsev, A. N.; Sönnichsen, F. D.; Hövener, J. Continuous Radio Amplification by Stimulated Emission of Radiation Using Parahydrogen Induced Polarization (PHIP-RASER) at 14 Tesla. *ChemPhysChem* **2020**, *21* (7), 667–672. https://doi.org/10.1002/cphc.201901056.

(9) Pravdivtsev, A. N.; Hövener, J.-B.; Schmidt, A. B. Frequency-Selective Manipulations of Spins Allow Effective and Robust Transfer of Spin Order from Parahydrogen to Heteronuclei in Weakly-Coupled Spin Systems. *ChemPhysChem* **2022**, *23* (3), e202100721. https://doi.org/10.1002/cphc.202100721.

(10) Salnikov, O. G.; Assaf, C. D.; Yi, A. P.; Duckett, S. B.; Chekmenev, E. Y.; Hövener, J.-B.; Koptyug, I. V.; Pravdivtsev, A. N. Modeling Ligand Exchange Kinetics in Iridium Complexes Catalyzing SABRE Nuclear Spin Hyperpolarization. *Anal. Chem.* **2024**, acs.analchem.4c01374. https://doi.org/10.1021/acs.analchem.4c01374.

(11) Pravdivtsev, A. N.; Yurkovskaya, A. V.; Zimmermann, H.; Vieth, H.-M.; Ivanov, K. L. Enhancing NMR of Insensitive Nuclei by Transfer of SABRE Spin Hyperpolarization. *Chem. Phys. Lett.* **2016**, *661*, 77–82. https://doi.org/10.1016/j.cplett.2016.08.037.

(12) Yi, A. P.; Salnikov, O. G.; Burueva, D. B.; Chukanov, N. V.; Chekmenev, E. Y.; Koptyug, I. V. Solvent Effects in Hyperpolarization of 15N Nuclei in [15N3]Metronidazole and [15N3]Nimorazole Antibiotics via SABRE-SHEATH**. *Anal. Sens.* **2024**, e202400045. https://doi.org/10.1002/anse.202400045.

(13) Knecht, S.; Hadjiali, S.; Barskiy, D. A.; Pines, A.; Sauer, G.; Kiryutin, A. S.; Ivanov, K. L.; Yurkovskaya, A. V.; Buntkowsky, G. Indirect Detection of Short-Lived Hydride Intermediates of Iridium N-Heterocyclic Carbene Complexes via Chemical Exchange Saturation Transfer Spectroscopy. *J. Phys. Chem. C* **2019**, *123* (26), 16288–16293. https://doi.org/10.1021/acs.jpcc.9b04179.

(14) Neese, F.; Wennmohs, F.; Becker, U.; Riplinger, C. The ORCA Quantum Chemistry Program Package. *J. Chem. Phys.* **2020**, *152* (22), 224108. https://doi.org/10.1063/5.0004608.
(15) Becke, A. D. A New Mixing of Hartree–Fock and Local Density-Functional Theories. *J. Chem. Phys.* **1993**, *98* (2), 1372–1377. https://doi.org/10.1063/1.464304.
(16) Lee, C.; Yang, W.; Parr, R. G. Development of the Colle-Salvetti Correlation-Energy Formula into a Functional of the Electron Density. *Phys. Rev. B* **1988**, *37* (2), 785–789. https://doi.org/10.1103/PhysRevB.37.785.
(17) Caldeweyher, E.; Bannwarth, C.; Grimme, S. Extension of the D3 Dispersion Coefficient Model. *J. Chem. Phys.* **2017**, *147* (3), 034112. https://doi.org/10.1063/1.4993215.
(18) Weigend, F.; Ahlrichs, R. Balanced Basis Sets of Split Valence, Triple Zeta Valence and Quadruple Zeta Valence Quality for H to Rn: Design and Assessment of Accuracy. *Phys. Chem. Chem. Phys.* **2005**, *7* (18), 3297. https://doi.org/10.1039/b508541a.
(19) Hellweg, A.; Hättig, C.; Höfener, S.; Klopper, W. Optimized Accurate Auxiliary Basis Sets for RI-MP2 and RI-CC2 Calculations for the Atoms Rb to Rn. *Theor. Chem. Acc.* **2007**, *117* (4), 587–597. https://doi.org/10.1007/s00214-007-0250-5.
(20) Weigend, F. Hartree–Fock Exchange Fitting Basis Sets for H to Rn †. *J. Comput. Chem.* **2008**, *29* (2), 167–175. https://doi.org/10.1002/jcc.20702.
(21) Barone, V.; Cossi, M. Quantum Calculation of Molecular Energies and Energy Gradients in Solution by a Conductor Solvent Model. *J. Phys. Chem. A* **1998**, *102* (11), 1995–2001. https://doi.org/10.1021/jp9716997.
